# Awareness and knowledge of dementia risk reduction among current and future health professionals: A survey study

**DOI:** 10.1002/alz.70781

**Published:** 2025-10-07

**Authors:** Dominique Paauw, Irene Heger, Dorothee Horstkötter, Sebastian Köhler, Kay Deckers

**Affiliations:** ^1^ Mental Health and Neuroscience Research Institute (MHeNs), Alzheimer Centre Limburg, Department of Psychiatry and Neuropsychology Maastricht University Maastricht the Netherlands; ^2^ Department of Health, Ethics and Society Maastricht University Maastricht the Netherlands

**Keywords:** awareness, dementia, lifestyle, prevention, risk factors, risk reduction

## Abstract

**INTRODUCTION:**

To lower future dementia incidence, there is an urgent need to implement dementia risk reduction strategies in routine care. Yet, it remains unclear whether health professionals possess sufficient knowledge.

**METHODS:**

A cross‐sectional survey was conducted among 368 current and 692 future (i.e., students) health professionals in the Netherlands, assessing awareness of dementia risk reduction, knowledge of dementia risk factors, and educational needs and barriers.

**RESULTS:**

Most professionals (79.1%) and students (54.1%) were aware of dementia risk reduction. Across both groups, highly educated individuals demonstrated greater awareness and better recognition of risk factors. Knowledge gaps existed within both groups regarding the contribution of hearing impairment, obesity, poor sleep, and chronic kidney disease. Both groups expressed interest in professional education on brain health promotion.

**DISCUSSION:**

These findings highlight the need for tailored educational modules to address knowledge gaps and equip professionals with the tools to discuss dementia risk reduction in routine care.

**Highlights:**

- Most professionals and students were aware of dementia risk reduction possibilities.
- Highly educated individuals demonstrated greater awareness and better recognition of dementia risk factors than those with lower education levels.
- Both professionals and students had knowledge gaps regarding specific dementia risk factors.
- Few students received comprehensive education on dementia risk reduction as part of their studies.
- The majority of professionals and students expressed interest in further (professional) education on improving brain health and dementia risk reduction.

## 1 BACKGROUND

Over the past decade, evidence has accumulated demonstrating the contribution of several lifestyle and cardiometabolic risk factors in the development of dementia.^1^, ^2^ Despite this growing body of research, there remains a lack of awareness among the general population regarding the potential of dementia risk reduction.^3^ In Europe, public awareness of the importance of lifestyle and general health with dementia risk has been the focus of several studies, but findings have been inconsistent, with awareness levels ranging from 34% to 70%.^4^, ^5^, ^6^, ^7^, ^8^ Furthermore, public understanding of the relationship between modifiable cardiometabolic risk factors, such as hypertension and hypercholesterolemia, and dementia remains limited.^9^ Increasing public knowledge on dementia risk and protective factors is a crucial first step toward more successful risk reduction strategies.^10^

Health professionals could play a pivotal role in informing the public about dementia risk reduction possibilities during patient encounters by supporting behavior change and offering appropriate referrals. However, similar to findings from the general population, several studies show that both practicing professionals and students in health‐related fields often have moderate to low awareness of dementia risk reduction and knowledge of modifiable dementia risk and protective factors.^11^, ^12^, ^13^, ^14^, ^15^, ^16^, ^17^ Furthermore, qualitative and survey studies have shown that general practitioners and other health professionals express a desire for more information and training on dementia risk reduction, including guidance and tools to communicate effectively about dementia risk and to integrate dementia risk reduction into routine primary care.^18^, ^19^

Given the potential of dementia risk reduction to lower future dementia incidence, it is crucial to educate current and future health professionals on dementia risk reduction strategies. This study aimed to assess the level of awareness regarding dementia risk reduction and knowledge of proposed modifiable dementia risk and protective factors among health professionals and students in the Netherlands, including those from various health disciplines and educational backgrounds. Additionally, the study sought to explore their needs and perceived barriers regarding educational programs and tools focused on dementia risk reduction.

## 2 METHODS

### 2.1 Study population and procedure

The target population of this cross‐sectional survey study was (1) health professionals from various disciplines (e.g., general practitioners [GPs], practice nurses, nurses, [dementia] case managers [professionals who coordinate care and support for individuals with a chronic illness (e.g, dementia) and their families], physical therapists, psychologists, dietitians) employed in the Netherlands; and (2) students enrolled in health‐related or adjacent programs (e.g., medicine, psychology, health sciences, nursing, social work, and pedagogical studies) in the Netherlands across different education levels (secondary vocational education, higher vocational education, and academic higher education).

Professionals and students were recruited between September 2023 and March 2024 via social media platforms, patient organizations/health‐care institutions, educational institutions, advertisements in newsletters from study associations and professional organizations, and personal networks from the research team. Additional details on the recruitment channels are provided in Supplementary File 1 in supporting information. After providing informed consent, participants completed the online survey anonymously via Qualtrics XM. Participation was voluntary and no financial compensation was offered. The English translation of the complete surveys for both professionals and students are provided in Supplementary Files 2 and 3 in supporting information.

### 2.2 Measurements

All participants (professionals and students) completed questions on demographics (age, sex, and education level), self‐reported knowledge of dementia, awareness of dementia risk reduction, knowledge of modifiable dementia risk/protective factors, and interest in receiving further education on dementia risk reduction through lifestyle modifications. The surveys were modified versions of an existing survey for studying awareness and knowledge of dementia risk reduction in the general population.^4^, ^5^, ^6^, ^7^, ^8^, ^20^

Education level of professionals was obtained by self‐assessment of the highest finalized degree, using six categories based on the Dutch education system, which were grouped into three levels: low, middle, and high. The education level of students was also obtained by self‐assessment of the highest finalized degree and categorized according to the Dutch education system as secondary vocational education, higher vocational education, or academic higher education. Professional discipline was grouped into four categories: physicians, nurses, allied health professionals, and other. A detailed categorization of job titles can be found in Supplementary File 4 in supporting information. Years of professional experience was obtained by a single item with four answer options: 0 to 5 years, 6 to 10 years, 11 to 20 years, or > 20 years. Academic discipline was obtained by using a single item with four answer options: health, behavior and society, education and upbringing, and other.

**RESEARCH IN CONTEXT**

1. **Systematic review**: We reviewed the literature on dementia risk reduction awareness among current and future health professionals, using traditional medical databases (e.g., PubMed). While existing research suggests that most health professionals and health students have poor to moderate awareness of dementia risk reduction, no studies have specifically examined their needs, wishes, or perceived barriers regarding educational programs and tools focused on dementia risk reduction.
2. **Interpretation**: Our findings suggest that awareness of the potential of dementia risk reduction is fairly good among professionals and moderate among students. Both professionals and students demonstrated considerable knowledge gaps regarding specific dementia risk factors, such as hearing impairment, obesity, poor sleep, and chronic kidney disease. A strong gradient was seen across educational backgrounds. Both groups expressed interest in receiving more information on dementia risk reduction.
3. **Future directions**: Our findings highlight the need to (further) educate professionals and student about the potential of dementia risk reduction. Developing online educational modules and tools to address dementia risk reduction may help fill existing knowledge gaps.

Self‐reported knowledge of dementia was assessed using a single item, consisting of a brief explanation of the condition followed by the question: “How much would you say you know about dementia?”. There were six answer options, which were dichotomized for analysis into “good” (comprising “excellent,” “good,” and “considerable”) and “poor” (comprising “poor,” “nothing at all,” and “I don't know”). The primary outcome was awareness of dementia risk reduction, assessed by the proportion of participants rejecting the statement “There is nothing one can do to reduce their risk of getting dementia.” The secondary outcome was knowledge of 15 proposed modifiable risk and protective factors for dementia, based on the recently updated LIfestyle for BRAin Health (LIBRA) dementia risk score.^2^, ^21^ Answers to statements on awareness and knowledge were coded as binary variables (aware/unaware) for analyses. There were six answer options: “agree strongly,” “agree,” “neither agree nor disagree,” “disagree,” and “disagree strongly”. The answer option “neither agree nor disagree” was coded as unaware. A sum score (range 0–15) was calculated as the average number of correctly identified factors, with each correctly identified factor coded as “aware” (1) and each incorrectly identified or unidentified factor coded as “unaware” (0).

Both professionals and students answered questions concerning their interest in lifestyle and brain health and preferred source(s) of information. Professionals were asked specific questions about their professional role, years of experience, whether they provide information on dementia risk reduction through lifestyle modification to their patients, the dementia risk and protective factors they address, and their use of informational materials. Additionally, professionals were asked about the perceived importance of professional education on dementia risk reduction, perceived barriers in clinical practice for educating about brain health promotion, and their interest in more information to improve brain health. Students answered specific questions about their education institution, academic discipline, and whether their curriculum included information on dementia risk reduction through lifestyle modifications. Students were also asked about the perceived relevance of dementia risk reduction to their studies, and their interest in more information to improve brain health.

### 2.3 Statistical analysis

Participants were excluded from the dataset if they did not meet the inclusion criteria (aged <16 or >70 years for professionals or aged <16 or >50 years for students) or discontinued the survey prior to answering the (primary) outcome measures. Independent samples *t*tests and one‐way analysis of variance tests were conducted to examine differences in awareness of dementia risk reduction and knowledge of dementia risk and protective factors based on sex, academic discipline (students), years of professional experience (professionals), and professional discipline (professionals). *χ* ^2^ tests were used to analyze associations between sociodemographic variables (age, sex, education level, academic discipline, years of professional experience, and professional discipline) and level of awareness, knowledge of dementia risk and protective factors, as well as needs, wishes, and barriers. Additionally, *χ* ^2^ tests were used to examine the association between self‐reported knowledge of dementia and awareness of dementia risk reduction. All analyses were performed using Stata 17.0 (StataCorp), with the level of statistical significance set at 0.05 in two‐tailed tests.

### 2.4 Ethical approval

All participants were informed about the study and provided digital informed consent before participating in the online survey. Ethical approval has been obtained from the medical ethics committee of Maastricht University Medical Centre+ (METC azM/UM; reference number 2023‐0026).

## 3 RESULTS

### 3.1 Demographics

The study sample consisted of 368 health professionals and 692 health students. Their characteristics are shown in Table 1.

**TABLE 1:** Sample characteristics of health professionals and students.

Sample characteristics	Professionals n = 368	Students n = 692
Age (years), mean (SD^a^ ^,^ ^b^)	47.8 (13.4)	24.0 (8.9)
Female, n (%)	325 (88.3%)	605 (87.4%)
Education level, n (%)		
Low	27 (7.3%)	
Middle	124 (33.7%)	
High	217 (59.0%)	
Secondary vocational education		449 (64.9%)
Higher vocational education		124 (17.9%)
Academic higher education		119 (17.2%)
Professional discipline, n (%)		
Physicians	11 (3.0%)	
Nurses	144 (39.1%)	
Paramedics	121 (32.9%)	
Other	92 (25.0%)	
Academic discipline, n (%)		
Health		475 (68.6%)
Behavior and society		143 (20.7%)
Education and upbringing		55 (8.0%)
Other		19 (2.8%)
Years of professional experience, n (%)		
0–5 years	141 (38.3%)	
6–10 years	46 (12.5%)	
11–20 years	71 (19.3%)	
> 20 years	110 (29.9%)	
Self‐reported knowledge of dementia, n (%)		
Good	348 (94.6%)	623 (90.0%)
Poor	20 (5.4%)	69 (10.0%)

*Note*: Percentages may not sum to 100% because of rounding errors.

^a^ Standard deviation (SD).

^b^ Age groups (years), *n* (%); professionals: 17–35 years: 78 (21.2%); 36–55 years: 155 (42.1%); 56–70 years: 135 (36.7%); students: 16–25 years: 524 (75.7%); 26–50 years: 168 (24.3%).

### 3.2 Awareness of dementia risk reduction

#### 3.2.1 Professionals

**FIGURE 1:**
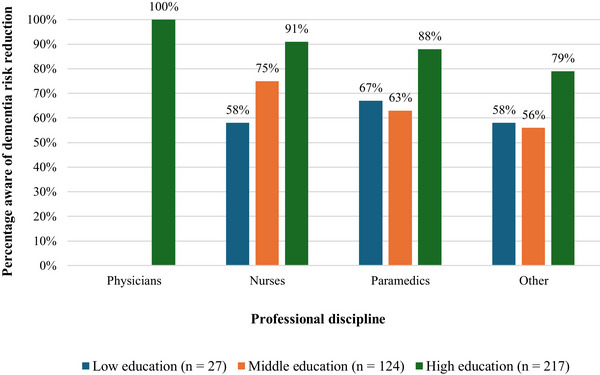
Awareness of dementia risk reduction among professionals. Differences in awareness of dementia risk reduction among professionals stratified by professional discipline and education level.

Most professionals (79.1%) were aware of the possibility of dementia risk reduction. Awareness was significantly higher among professionals with a higher level of education compared to those with lower education levels (low: 59.3%, middle: 68.6%, high: 87.6%; *χ* ^2^ [2] = 24.15, *p* ≤ 0.001), as is shown in Figure 1. Awareness also varied significantly across professional disciplines (*χ* ^2^ [3] = 11.20, *p* = 0.011), with physicians demonstrating the highest awareness (100%), followed by allied health professionals (84.3%), nurses (79.9%), and other health professionals (68.5%). Additionally, professionals who self‐reported to have good knowledge of dementia were significantly more likely to be aware of dementia risk reduction than those with poor self‐reported dementia knowledge (80.2% vs. 60.0%; *χ* ^2^ [1]) = 4.65, *p* = 0.031). No significant differences in awareness were observed between age groups, sex, or across years of professional experience (see Supplementary File 5 for detailed statistics).

#### 3.2.2 Students

**FIGURE 2:**
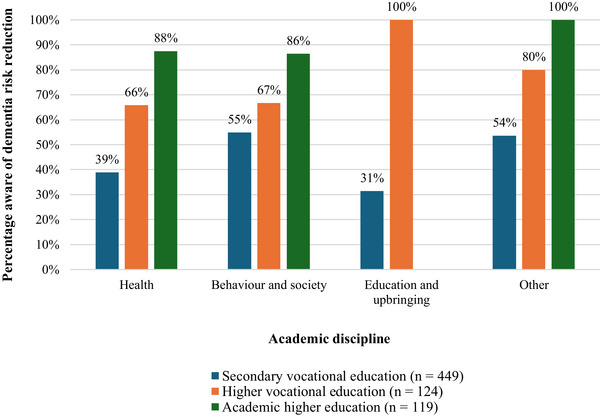
Awareness of dementia risk reduction among students. Differences in awareness of dementia risk reduction among students stratified by academic discipline and education level.

More than half of the students (54.1%) were aware of dementia risk reduction. Awareness was significantly higher in academic higher education (87.4%) compared to higher vocational education (67.7%) and secondary vocational education (41.4%; *χ* ^2^ [2] = 91.45, *p* ≤ 0.001), as shown in Figure 2. Older students were significantly more aware than younger students (aged 26–50: 65.5% vs. aged 16–25: 50.4%; *χ* ^2^ [1] = 11.67, *p* ≤ 0.001). There were no significant differences in awareness observed by sex or self‐reported knowledge levels of dementia (see Supplementary File 5 for detailed statistics).

### 3.3 Knowledge of dementia risk and protective factors

#### 3.3.1 Professionals

**FIGURE 3:**
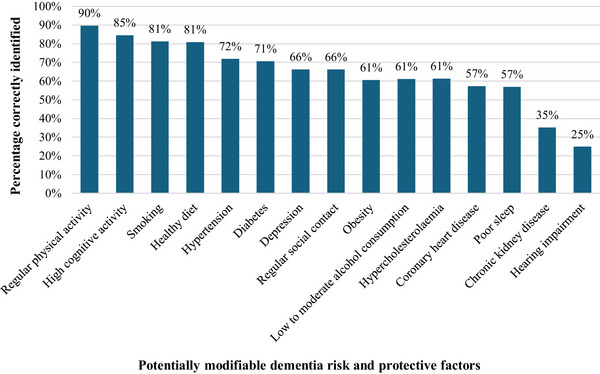
Knowledge of dementia risk and protective factors among professionals. Percentage of correctly identified dementia risk and protective factors among professionals (*n* = 368).

Regular physical activity (89.7%) was most frequently identified as protective against dementia, followed by high cognitive activity (84.5%). Smoking was most frequently recognized as a risk factor for dementia (81.3%). In contrast, poor sleep (56.9%), chronic kidney disease (35.2%), and hearing impairment (24.9%) were the least frequently identified risk factors (Figure 3). On average, professionals correctly identified 9.8 (standard deviation [SD] = 3.7) out of 15 proposed dementia risk and protective factors. Highly educated professionals recognized more risk and protective factors on average compared to lower educated professionals (low: 7.0, middle: 9.0, high: 10.5; *F*[2, 342] = 15.80, *p* = 0.002). No significant differences were found between age groups, sex, professional disciplines, years of professional experience, or self‐reported knowledge of dementia (see Supplementary File 5 for detailed statistics).

#### 3.3.2 Students

**FIGURE 4:**
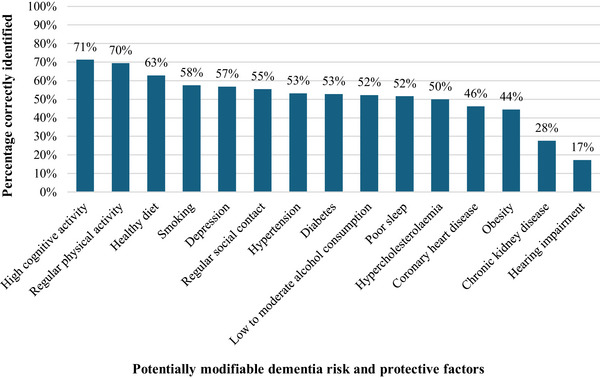
Knowledge of dementia risk and protective factors among students. Percentage of correctly identified dementia risk and protective factors among students (*n* = 692).

Students most frequently identified high cognitive activity (71.3%), regular physical activity (69.5%), and healthy diet (62.9%) as protective against dementia. On the other hand, obesity (44.5%), chronic kidney disease (27.7%), and hearing impairment (17.3%) were the least frequently identified risk factors for dementia (Figure 4). On average, students recognized 7.7 (SD = 4.2) out of 15 proposed dementia risk and protective factors. There were significant differences in the average number of correctly identified risk and protective factors between age groups (26–50 years: 8.7 vs. 16–25 years: 7.4; *t* [663]: –3.57, *p* ≤ 0.001), education levels (secondary vocational education: 6.6, higher vocational education: 9.0, academic higher education: 10.5; *F*[2, 662] = 51.77, *p* ≤ 0.001), academic disciplines (health: 8.0, behavior and society: 7.3, education and upbringing: 6.0, other: 7.6; *F*[3,661] = 4.00, *p* = 0.008), and reported knowledge levels of dementia (good: 7.9 vs. poor: 6.1; *t* [663]: 3.21, *p* = 0.001). No significant differences were found between sex (see Supplementary File 5 for detailed statistics).

### 3.4 Interest for information on brain health promotion

#### 3.4.1 Professionals

The majority (61.7%) of professionals stated they would like to receive more information on brain health promotion. There were no significant differences in interest in more information between age groups, sex, educational levels, professional disciplines, or years of professional experience (see Supplementary File 5 for detailed statistics).

#### 3.4.2 Students

More than half (55.5%) of the students stated they would like to receive more information on brain health promotion, with highly educated students being more interested compared to lower educated students (secondary vocational education: 49.5%, higher vocational education: 65.5%, academic higher education: 68.1%; *χ* ^2^ [4] = 22.54, *p* ≤ 0.001). Also, health students were more interested in receiving such information than students of other academic disciplines (health: 57.9%, behavior and society: 52.9%, education and upbringing: 38.9%, other: 63.2%; *χ* ^2^ [6] = 16.70, *p* = 0.010). There were no significant differences between age groups or sex (see Supplementary File 5 for detailed statistics).

### 3.5 Education on dementia risk reduction

#### 3.5.1 Professionals

The majority of professionals (61.9%) considered professional education on dementia risk reduction through lifestyle modifications as important in their field, while 26.2% viewed it as potentially important, and 12% as not important. Allied health professionals were more likely to have interest in professional education compared to other professional disciplines (physicians: 54.6%, nurses: 54.3%, allied health professionals: 75.7%, other: 56.3%; *χ* ^2^ [6] = 20.26, *p* = 0.002). Most professionals (57.9%) expressed interest in receiving additional professional education on dementia risk reduction. No significant differences were found in interest across professional disciplines or across years of professional experience (see Supplementary File 5 for more detailed statistics).

#### 3.5.2 Students

More than half of the students (55.1%) stated to have received some kind of information on reducing dementia risk reduction through lifestyle modifications during their studies, with 12.8% of the students indicating they received comprehensive information. Health students reported more often to have received information on dementia risk reduction compared to students of other fields (health: 61.9%, behavior and society: 46.7%, education and upbringing: 18.5%, other: 57.9%; *χ* ^2^ [3] = 41.58, *p* ≤ 0.001). Most students (78.2%) considered it important to receive such information during their studies, 16.0% as potentially important, and 5.8% as not important. The perceived importance of receiving such information varied significantly across academic disciplines (*χ* ^2^ [6] = 185.07, *p* ≤ 0.001): 83.6% of health students considered it important, followed by students in behavior and society (82.8%), other health‐related fields (73.7%), and education and upbringing (24.1%). Students who did not consider this information important provided various reasons, including its perceived irrelevance to them (education and upbringing: 87.5%, health: 57.1%, behavior and society: 40.0%, other: 100%), lower priority for them (education and upbringing: 20.8%, health: 14.3%, behavior and society: 20%, other: 0%), and insufficient time or opportunity to discuss the topic during their studies (education and upbringing: 16.7%, health: 28.6%, behavior and society: 0%, other: 0%).

### 3.6 Current information dissemination on dementia risk reduction

Most professionals (61.4%) reported providing patients or clients with information on reducing dementia risk through lifestyle modifications, but only 19.5% did so regularly. Physicians and allied health professionals were more likely to discuss dementia risk reduction compared to other professional disciplines (physicians: 72.7%, nurses: 61.1%, allied health professionals: 70.9%, other: 47.6%; *χ* ^2^ [3] = 11.42, *p* = 0.010). No significant differences were observed across years of professional experience (see Supplementary File 5 for detailed statistics). The most frequently discussed dementia risk factors among professionals who provided dementia risk reduction information were healthy diet (11.5%), low cognitive activity (11%), and low social contact (11%), whereas hypercholesterolemia (4.5%), hearing impairment (4.5%), and chronic kidney disease (2.5%) were the least discussed. Among professionals who provided dementia risk reduction information, 65.4% used educational materials (e.g., brochures or website of the Dutch Alzheimer's Association). Professionals who reported to not provide dementia risk reduction information reported various reasons, including its perceived irrelevance for their profession (16.2%), lack of knowledge (15.2%), and the absence of applicable guidelines (15.2%).

## 4 DISCUSSION

This study evaluated the awareness of dementia risk reduction and knowledge of proposed modifiable risk and protective factors among current and future health professionals, as well as their current practices, interests, and perceived barriers regarding education on brain health promotion. Professionals demonstrated better awareness about possibilities to reduce the risk of dementia and identified more risk and protective factors than students. In both groups, awareness and knowledge of risk and protective factors increased with higher educational attainment. While most professionals and students were aware of the potential of dementia risk reduction and recognized its relevance in both clinical practice and professional education, substantial knowledge gaps were observed in both groups. Most professionals and students expressed an interest in further education on dementia risk reduction.

### 4.1 Awareness and knowledge of risk and protective factors

Professionals demonstrated a higher level of awareness and identified more dementia risk and protective factors than students, aligning with findings from previous studies.^11^, ^17^ More highly educated professionals were most aware of the potential of dementia risk reduction, suggesting that further training on this subject is needed among professionals with lower levels of education. While 90% of students reported good general knowledge of dementia, awareness of dementia risk reduction was relatively low, especially among those with lower education levels. These findings highlight the importance of integrating dementia risk reduction content across all levels of education, including more practical‐oriented training programmes.

The most frequently recognized protective factors among both students and professionals were high cognitive activity, regular physical activity, and healthy diet, aligning with prior studies.^11^, ^13^ Notably, these protective factors are also the most frequently identified by the general public.^4^, ^5^, ^6^, ^7^, ^20^ Yet, significant knowledge gaps were observed regarding the contribution to dementia risk of chronic kidney disease, hearing impairment, obesity, and poor sleep, with recognition percentages <65%. Limited awareness of dementia risk factors, especially cardiometabolic risk factors, has been previously reported among students.^11^, ^16^ The low recognition of hearing impairment and poor sleep as risk factors for dementia may be attributed to their relatively recent identification as candidate modifiable risk factors.^1^, ^2^

### 4.2 Education on dementia risk reduction and interest in more information

According to the World Health Organization, strengthening the ability of health professionals to deliver evidence‐based information on dementia risk reduction could play a key role in reducing dementia risk or slowing its progression.^22^ However, our findings indicate that <20% of professionals regularly provide patients or clients with information on dementia risk reduction during routine practice. Nonetheless, more than half of professionals indicated a willingness to receive further professional education on this topic, and this interest was consistent across disciplines and years of working experience. This broad interest highlights the importance of integrating dementia risk reduction into the training and further professional education of all health disciplines, ensuring that professionals across the whole health spectrum are equipped to address this pressing health issue. Among students, more than three quarters considered it important to learn about dementia risk reduction during their studies. Among those who did not, the most commonly reported reason was its perceived irrelevance to their study, particularly among students outside the health discipline. Other reasons included the lower priority of the topic and limited time or opportunity to cover the topic during their studies. While most students reporting having received some information on dementia risk reduction during their studies, only a small proportion indicated that they received comprehensive coverage on this topic, and more than half were interested in further education on this topic. These findings highlight the need for education strategies that are tailored to the specific disciplines and roles of both professionals and students.

### 4.3 Strengths and weaknesses

This study has several strengths. To our knowledge, it is the first to report on the awareness and knowledge of dementia risk reduction among both current and future health professionals using a large and diverse sample. The inclusion of a large and heterogeneous sample of both practicing professionals and students across various professional and academic disciplines enhances the generalizability of the findings and allows for comparisons between subgroups. Furthermore, the surveys used were modified versions of an existing survey that has been administered in various populations to assess awareness and knowledge of dementia risk reduction.^4^, ^5^, ^6^, ^7^, ^8^, ^20^ However, some limitations should be considered. Selection bias may have occurred due to recruitment through social media platforms and newsletters associated with professional and academic organizations, potentially excluding individuals who do not engage with these channels. Furthermore, the distribution of education levels in this sample does not reflect that of the general Dutch population. Specifically, there was an overrepresentation of students from secondary vocational education, leading to a predominance of lower educated students, while the professional sample consisted largely of highly educated individuals. This imbalance may have attenuated differences between education levels and between professionals and students. While there is evidence of a modest link between treating certain risk factors (e.g., hypertension) and reduced cognitive decline or dementia risk, robust causal evidence is still lacking for most LIBRA factors. Nonetheless, addressing dementia risk and protective factors is recommended, as it can positively impact overall health and a range of health conditions.

### 4.4 Knowledge gaps and future directions

Health professionals play a critical role in educating patients about dementia risk reduction. As key entry points into both care and community services, their awareness and knowledge of dementia risk reduction can help embed this topic into routine practice. For instance, Van Asbroeck et al.^23^ demonstrated that integrating dementia risk reduction education into GP settings (within existing chain care for diabetes or cardiovascular risk factors) is both feasible and well received by patients and providers. When professionals possess awareness and knowledge of dementia risk reduction, they are better able to guide, advise, and treat individuals seeking information. Although chronic conditions such as hypertension, hypercholesterolemia, obesity, and chronic kidney disease are already subject to medical treatment, explicitly communicating their link to dementia risk/brain health may enhance patient motivation, improve adherence to treatments, and encourage the adoption of preventive lifestyle modifications.^18^, ^23^, ^24^ This, in turn, enhances the impact of broader efforts, such as public awareness campaigns, and may foster wider support for population‐level approaches to dementia risk reduction.^25^ However, the pronounced gradient in awareness and the observed knowledge gaps regarding certain dementia risk factors, alongside the expressed interest in more information, underscore the pressing need for improved education. This applies to both professional training and formal education curricula, particularly considering the expected rise in dementia cases worldwide. To address these challenges, we recommend the development and implementation of tailored education modules for both professionals and students. For professionals, these modules should incorporate practical tools (e.g., conversation starters) and role‐specific examples to support the integration of dementia risk reduction into daily practice. For students, early inclusion of dementia risk reduction within the curriculum, with an emphasis on its cross‐disciplinary relevance, could foster greater engagement. A comprehensive and interdisciplinary approach to dementia risk reduction education will be essential to ensure that both current and future health professionals are well prepared to help lower future dementia incidence.

## 5 CONCLUSION

The present study indicates that awareness of dementia risk reduction is fairly good among professionals, but moderate among students, especially with lower education background and more practical training. Considerable knowledge gaps exist in both professionals and students regarding certain proposed modifiable dementia risk factors, as comprehensive education on the topic is currently lacking. The majority of both groups expressed a need for more information on dementia risk reduction. Developing education materials to address these needs is important to ensure that health professionals are well equipped to provide information on brain health and support their patients in understanding how cardiometabolic or lifestyle factors may influence their risk of developing dementia.

## CONFLICT OF INTEREST STATEMENT

The authors declare no conflicts of interest. Author disclosures are available in the supporting information.

## CONSENT STATEMENT

All participants provided informed consent.

## Supporting information



Supporting Information

Supporting Information
