# Supplementary material for: Awareness and knowledge of dementia risk reduction among current and future health professionals: A survey study
Source: Alzheimers Dement. 2025 Oct 7;21(10):e70781. doi: 10.1002/alz.70781 (PMC12504045; doi:10.1002/alz.70781)
Supplement: Supplementary file 1 — Supporting Information [file ALZ-21-e70781-s001.docx]

**Supplementary File 1: Overview of recruitment channels used**

| **Channel** | **Target group** | **Name** |
| --- | --- | --- |
| Social media | Students and professionals | LinkedIn, Instagram, Facebook (social media account from Alzheimer Centrum Limburg) |
| Study associations | Students | MSV Santé (Health sciences, Maastricht University), MSV Pulse (Medicine, Maastricht University), SV HALO (Health care psychology, Radboud University) |
| Professional organisations | Professionals | Netherlands Institute of Psychologists, Netherlands Association for Neuropsychology, Netherlands Society of Neurology, NU’91 (Dutch professional organisation for nurses and care workers), Dutch Association of Practice Support Workers and Practice Nurses, |
| Personal networks | Students and professionals | Shared by researchers |
| Patient organisations/charities | Students and professionals | Dutch Brain Foundation, Alzheimer Netherlands |
| Healthcare institutions | Students and professionals | Maastricht University Medical Center+, Sevagram, Meander, Envida, De Zorggroep |
| Educational institutions | Students | Maastricht University, Zuyd University of Applied Sciences, VISTA College, Gilde Opleidingen, Inholland University of Applied Sciences, Windesheim University of Applied Sciences |

**Supplementary File 2: English translation of the dementia awareness survey for professionals**

Demographics

1. How old are you?

*[Text field for number between 16 and 99]*

1. What is your gender?

- Male
- Female
- Other

1. What is your highest finalized degree of education?

*[Six categories according to the Dutch education system, categorized into low, middle, and high]*

1. What is your job title?

- General practitioner
- Practice nurse
- Nurse
- Case manager (dementia)
- Physical therapist
- Occupational therapist
- Other, namely: *[open text field]*

1. How many years of experience do you have in this role?

- 0 to 5 years
- 6 to 10 years
- 11 to 20 years
- More than 20 years

Dementia knowledge

1. Dementia describes a set of symptoms including loss of memory, sudden mood swings, not remembering who people are, and having trouble finding your words. Alzheimer’s disease is one form of dementia. How much would you say you know about dementia?

- Excellent
- Good
- Considerable
- Poor
- Nothing at all
- I don’t know

Dementia risk awareness

Please indicate below how much you agree or disagree with the following statement.

1. ‘There is nothing one can do to reduce their risk of getting dementia’

- Agree strongly
- Agree
- Neither agree nor disagree
- Disagree
- Disagree strongly

1. ‘High blood pressure increases your chances of getting dementia’

- Agree strongly
- Agree
- Neither agree nor disagree
- Disagree
- Disagree strongly

1. ‘Smoking increases your chances of getting dementia’

- Agree strongly
- Agree
- Neither agree nor disagree
- Disagree
- Disagree strongly

1. ‘No or moderate alcohol use lowers your chances of getting dementia’

- Agree strongly
- Agree
- Neither agree nor disagree
- Disagree
- Disagree strongly

1. ‘Regular physical activity lowers your chances of getting dementia’

- Agree strongly
- Agree
- Neither agree nor disagree
- Disagree
- Disagree strongly

1. ‘Depression increases the chances of getting dementia’
   - Agree strongly
   - Agree
   - Neither agree nor disagree
   - Disagree
   - Disagree strongly
2. ‘Diabetes increases the chances of getting dementia’
   - Agree strongly
   - Agree
   - Neither agree nor disagree
   - Disagree
   - Disagree strongly
3. ‘Being overweight increases the chances of getting dementia’
   - Agree strongly
   - Agree
   - Neither agree nor disagree
   - Disagree
   - Disagree strongly
4. ‘A mentally active lifestyle lowers the chances of getting dementia’
   - Agree strongly
   - Agree
   - Neither agree nor disagree
   - Disagree
   - Disagree strongly
5. ‘Heart disease increases the chances of getting dementia’
   - Agree strongly
   - Agree
   - Neither agree nor disagree
   - Disagree
   - Disagree strongly
6. ‘Kidney disease increases the chances of getting dementia’
   - Agree strongly
   - Agree
   - Neither agree nor disagree
   - Disagree
   - Disagree strongly
7. ‘High cholesterol increases the chances of getting dementia’
   - Agree strongly
   - Agree
   - Neither agree nor disagree
   - Disagree
   - Disagree strongly
8. ‘Healthy diet lowers the chances of getting dementia’
   - Agree strongly
   - Agree
   - Neither agree nor disagree
   - Disagree
   - Disagree strongly
9. ‘Regular contact with friends, family or colleagues lowers the chances of getting dementia’
   - Agree strongly
   - Agree
   - Neither agree nor disagree
   - Disagree
   - Disagree strongly
10. ‘Poor sleep increases the chances of getting dementia’
    - Agree strongly
    - Agree
    - Neither agree nor disagree
    - Disagree
    - Disagree strongly
11. ‘Impaired hearing increases the chances of getting dementia’
    - Agree strongly
    - Agree
    - Neither agree nor disagree
    - Disagree
    - Disagree strongly
12. Which of the factors listed below do you think are the three most important factors in the development of dementia?

*Three answer options*

- High blood pressure
- Smoking
- Excessive alcohol use
- Being physically active
- Depression
- Diabetes
- Obesity (overweight)
- Being mentally inactive
- Heart disease
- Kidney disease
- High cholesterol
- Unhealthy diet
- Little contact with friends, family or colleagues
- Poor sleep
- Impaired hearing

Needs, wishes and barriers

1. Would you be interested in information on how to improve your own brain health?

- Yes
- No
- Maybe

*[If answer is Yes, go to question 2]*

1. In the case that you would like to know more about your own brain health, what information channels would you use to find this information?

*Select all that apply*

- Internet (through a search)
- General practitioner or practice nurse
- Website of the GGD
- Website of the Dutch Alzheimer’s Association
- Library
- Other, namely: *[open text field]*
- None of the above
- I don’t know
- I prefer not to answer

1. In the case that you would decide to work on improving our own brain health, what could be an obstructing factor for you?

*Select all that apply*

- Lack of knowledge
- Lack of time
- Financial reasons
- Lack of motivation
- Difficult to organize
- Health problems
- Other, namely: *[open text field]*
- None of these
- I don’t know
- I prefer not to answer

Information about dementia risk reduction during the study

1. In your current role, do you provide information to your patients/clients about reducing the risk of dementia through lifestyle modifications (when applicable)?

- Yes, regularly
- Yes, sometimes
- No, never

*[If answer is Yes, go to question 2]*

*[If answer is No, go to question 5]*

1. What do you provide information on?

*Select all that apply*

- High blood pressure
- Smoking
- Low to moderate alcohol use
- Being physically active
- Depression
- Diabetes
- Obesity (overweight)
- Being mentally inactive
- Heart disease
- Kidney disease
- High cholesterol
- Healthy diet
- Regular contact with friends, family or colleagues
- Poor sleep
- Impaired hearing
- Other, namely: *[open text field]*

1. Do you use educational materials (such as a brochure or website) when providing information on reducing the risk of dementia?

- Yes, for most patients/clients
- Yes, for some patients/clients
- Yes, but only if patients/clients ask for it
- No

*[If answer is Yes, go to question 4]*

*[If answer is No, go to question 5]*

1. Which educational materials do you use?

*[Open text field]*

1. Why not?

*Select all that apply*

- I don’t know enough about this topic
- This topic is not relevant for my patients/clients
- I think that my patients/clients are not interested in this information
- It’s difficult to discuss this without making patients/clients feel blamed
- This topic has low priority
- There is insufficient time/opportunity to discuss this
- There is insufficient scientific evidence on the effectiveness
- There are no guidelines to use

Desire for professional education

1. Do you think that professional education on reducing the risk of dementia could be important within your field?

- Yes
- Maybe
- No

1. Would you be interested in professional education on reducing the risk of dementia through lifestyle modifications?

- Yes
- No
- Maybe

Follow-up research

1. Might we approach you for follow-up research?

- Yes, I give permission (please leave your email address): *[open text field]*
- No, I would rather not

End

Thank you very much for filling in this questionnaire. If you have any remarks or questions regarding this study, please contact us via [*email address*].

**NOTE**

If you are interested to use this survey, please contact: ([kay.deckers@maastrichtuniversity.nl](mailto:kay.deckers@maastrichtuniversity.nl)).

**Supplementary File 3: English translation of the dementia awareness survey for students**

Demographics

1. Are you 16 years or older?

- Yes
- No

*[15]*

1. How old are you?

*[Text field for number between 16 and 99]*

1. What is your gender?

- Male
- Female
- Other

1. What is your zip code? Enter only the four digits of your postal code.

*[Text field for four numbers]*

1. At what level are you currently pursuing education?

- Secondary vocational education
- Higher vocational education
- Academic higher education

*[If answer is secondary vocational education, go to question 6]*

*[If answer is higher vocational education, go to question 8]*

*[If answer is academic higher education, go to question 10]*

1. At which organisation are you following your secondary vocational education?

*[List of Dutch secondary vocational education institutions]*

1. In which sector are you following your secondary vocational education?

- Behaviour and society (such as: disability care attendant, maternity nurse, social service provider)
- Health (such as: nursing, optician, pharmacy assistant, physician assistant, dental assistant, personal healthcare assistant)
- Education and upbringing (such as: teaching assistant, childcare pedagogical assistant)
- Other, namely: *[open text field]*

1. At which organisation do you follow your higher vocational education?

*[List of Dutch higher vocational education institutions]*

1. In which sector do you follow your higher vocational education?

- Behaviour and society (such as: applied psychology, social work, applied gerontology, social work and service)
- Health (such as: occupational therapy, physical therapy, speech therapy, obstetrics, nursing, nutrition and dietetics)
- Education and upbringing (such as: teacher training, pedagogy, education science)
- Other, namely: *[open text field]*

1. At which organisation are you following your academic higher education?

*[List of Dutch academic higher education institutions]*

1. In which sector do you follow your academic higher education?

- Behaviour and society (such as: psychology, cultural anthropology, sociology, social studies)
- Health (such as: medicine, health sciences, pharmaceutical sciences, dentistry)
- Education and upbringing (such as: university teacher training, pedagogy and education science)
- Other, namely: *[open text field]*

1. Mention the name of your education here:

*[Open text field]*

1. How many years have you been following this education?

- Less than 1 year
- 1 or 2 years
- 3 or 4 years
- Longer than 4 years

Dementia knowledge

1. Dementia describes a set of symptoms including loss of memory, sudden mood swings, not remembering who people are, and having trouble finding your words. Alzheimer’s disease is one form of dementia. How much would you say you know about dementia?

- Excellent
- Good
- Considerable
- Poor
- Nothing at all
- I don’t know

Dementia risk awareness

Please indicate below how much you agree or disagree with the following statement.

1. ‘There is nothing one can do to reduce their risk of getting dementia’

- Agree strongly
- Agree
- Neither agree nor disagree
- Disagree
- Disagree strongly

1. ‘High blood pressure increases your chances of getting dementia’

- Agree strongly
- Agree
- Neither agree nor disagree
- Disagree
- Disagree strongly

1. ‘Smoking increases your chances of getting dementia’

- Agree strongly
- Agree
- Neither agree nor disagree
- Disagree
- Disagree strongly

1. ‘No or moderate alcohol use lowers your chances of getting dementia’

- Agree strongly
- Agree
- Neither agree nor disagree
- Disagree
- Disagree strongly

1. ‘Regular physical activity lowers your chances of getting dementia’

- Agree strongly
- Agree
- Neither agree nor disagree
- Disagree
- Disagree strongly

1. ‘Depression increases the chances of getting dementia’
   - Agree strongly
   - Agree
   - Neither agree nor disagree
   - Disagree
   - Disagree strongly
2. ‘Diabetes increases the chances of getting dementia’
   - Agree strongly
   - Agree
   - Neither agree nor disagree
   - Disagree
   - Disagree strongly
3. ‘Being overweight increases the chances of getting dementia’
   - Agree strongly
   - Agree
   - Neither agree nor disagree
   - Disagree
   - Disagree strongly
4. ‘A mentally active lifestyle lowers the chances of getting dementia’
   - Agree strongly
   - Agree
   - Neither agree nor disagree
   - Disagree
   - Disagree strongly
5. ‘Heart disease increases the chances of getting dementia’
   - Agree strongly
   - Agree
   - Neither agree nor disagree
   - Disagree
   - Disagree strongly
6. ‘Kidney disease increases the chances of getting dementia’
   - Agree strongly
   - Agree
   - Neither agree nor disagree
   - Disagree
   - Disagree strongly
7. ‘High cholesterol increases the chances of getting dementia’
   - Agree strongly
   - Agree
   - Neither agree nor disagree
   - Disagree
   - Disagree strongly
8. ‘Healthy diet lowers the chances of getting dementia’
   - Agree strongly
   - Agree
   - Neither agree nor disagree
   - Disagree
   - Disagree strongly
9. ‘Regular contact with friends, family or colleagues lowers the chances of getting dementia’
   - Agree strongly
   - Agree
   - Neither agree nor disagree
   - Disagree
   - Disagree strongly
10. ‘Poor sleep increases the chances of getting dementia’
    - Agree strongly
    - Agree
    - Neither agree nor disagree
    - Disagree
    - Disagree strongly
11. ‘Impaired hearing increases the chances of getting dementia’
    - Agree strongly
    - Agree
    - Neither agree nor disagree
    - Disagree
    - Disagree strongly
12. Which of the factors listed below do you think are the three most important factors in the development of dementia?

*Maximum of three answer options*

- High blood pressure
- Smoking
- Excessive alcohol use
- Being physically active
- Depression
- Diabetes
- Obesity (overweight)
- Being mentally inactive
- Heart disease
- Kidney disease
- High cholesterol
- Unhealthy diet
- Little contact with friends, family or colleagues
- Poor sleep
- Impaired hearing

Needs, wishes and barriers

1. Would you be interested in information on how to improve your own brain health?

- Yes
- No
- Maybe

*[If answer is Yes, go to question 2]*

1. In the case that you would like to know more about your own brain health, what information channels would you use to find this information?

*Select all that apply*

- Internet (through a search)
- General practitioner or practice nurse
- Website of the GGD
- Website of the Dutch Alzheimer’s Association
- Library
- Other, namely: *[open text field]*
- None of the above
- I don’t know
- I prefer not to answer

1. In the case that you would decide to work on improving our own brain health, what could be an obstructing factor for you?

*Select all that apply*

- Lack of knowledge
- Lack of time
- Financial reasons
- Lack of motivation
- Difficult to organize
- Health problems
- Other, namely: *[open text field]*
- None of these
- I don’t know
- I prefer not to answer

Information about dementia risk reduction during study

1. Was any information given during your study about reducing the risk of dementia through lifestyle modifications?

- Yes, comprehensive information
- Yes, but few
- No, never

*[If answer is Yes, go to question 2]*

1. What factors relevant to reducing the risk of dementia did you receive information about?

*Select all that apply*

- High blood pressure
- Smoking
- Low to moderate alcohol use
- Being physically active
- Depression
- Diabetes
- Obesity (overweight)
- Being mentally inactive
- Heart disease
- Kidney disease
- High cholesterol
- Healthy diet
- Regular contact with friends, family or colleagues
- Poor sleep
- Impaired hearing
- Other, namely: *[open text field]*

Desire for education on dementia risk reduction

1. Do you think that discussing lowering the risk of dementia is important within your study?

- Yes
- Maybe
- No

*[If answer is Yes or Maybe, go to question 3]*

*[If answer is No, go to question 2]*

1. Why not?

*Select all that apply*

- I don’t know enough about this to make a judgement
- This topic is not relevant to my studies
- I’m not interested in this information
- This topic has a lower priority for me
- There is insufficient time/opportunity to discuss this
- There is insufficient scientific evidence for this
- There are no guidelines to use
- Other, namely: *[open text field]*

Follow-up research

1. Might we approach you for follow-up research?

- Yes, I give permission (please leave your email address): *[open text field]*
- No, I would rather not

End

Thank you very much for filling in this questionnaire. If you have any remarks or questions regarding this study, please contact us via [*email address*].

**NOTE**

If you are interested to use this survey, please contact: ([kay.deckers@maastrichtuniversity.nl](mailto:kay.deckers@maastrichtuniversity.nl)).

**Supplementary File 4: Overview of professional discipline categorization**

| **Professional discipline** | **Job title** |
| --- | --- |
| Physicians | General practitioner, medical doctor in geriatrics medicine, geriatric medicine specialist trainee, geriatric psychiatrics |
| Nurses | Nurse (specialist), practice nurse, nursing assistant, care assistant, community nurse, specialised geriatric care assistant |
| Paramedics | Chronic care nurse in primary care, mental health nurse in primary care, doctor’s assistant, physical therapist, occupational therapist, dietitian, speech therapist, (clinical) psychologist, neuropsychologist, behavioural specialist, pharmacy technician, operating room assistant, pulmonary function technologist |
| Other | (Specialised) social worker, home care assistant, (informal) caregiver, welfare worker, personal/individual/group support worker, care/ambulatory support worker, care companion, psychodiagnostic assistant, director, team leader/manager, (elderly) care coordinator/manager, policy advisor, volunteer, information analyst, health scientist, music therapist, community support worker, nursing lecturer, educational material developer, clinical educator, client confidential advisor, communications officer, physical activity professional, specialised mediation team, hostess, chain coordinator, medical clown, fitness instructor, medical consultant, activity coach/coordinator/therapist, occupational health case manager |

**Supplementary File 5: Overview of non-significant comparisons**

| **Topic** | **Sample** | **Variable** | **Groups** | **Test statistic** | **p-value** |
| --- | --- | --- | --- | --- | --- |
| **Awareness of dementia risk reduction** | Professionals | Age group | 17–35 years: 78.2%  36–55 years: 83.2%  56–70 years: 74.8% | χ² (2) = 3.13 | 0.209 |
|  |  | Sex | Men: 81.4% Women: 78.8% | χ² (1) = 0.16 | 0.691 |
|  |  | Years of professional experience | 0–5 years: 77.3%  6–10 years: 93.5%  11–20 years: 80.3%  ≥20 years: 74.6% | χ² (3) = 7.46 | 0.059 |
|  | Students | Gender | Men: 60.7%  Women: 53.1%  Other: 66.7% | χ² (2) = 1.93 | 0.380 |
|  |  | Self-reported knowledge | Good: 54.3%  Poor: 52.2% | χ² (1) = 0.11 | 0.742 |
| **Knowledge of dementia risk/protective factors** | Professionals | Age group | 17–35 years: 10.4  36–55 years: 9.9  56–70 years: 9.3 | F(2, 342) = 2.15 | 0.118 |
|  |  | Sex | Men: 10.5  Women: 9.7 | t(343) = 1.29 | 0.199 |
|  |  | Professional discipline | Physicians: 11.3  Nurses: 9.7  Allied health professionals: 10.2  Other: 9.1 | F(3, 341) = 2.04 | 0.108 |
|  |  | Years of professional experience | 0–5 years: 9.8  6–10 years: 10.6  11-20 years: 10.0  >20 years: 9.2 | F(3, 341) = 1.76 | 0.113 |
|  |  | Self-reported knowledge of dementia | Good: 9.8  Poor: 9.0 | t(343) = 0.899 | 0.369 |
|  | Students | Gender | Men: 9.1  Women: 8.3  Other: 6.3 | F(2, 662) = 1.52 | 0.139 |
| **Interest for information on brain health promotion** | Professionals | Age group | 17–35 years: 60.0%  36–55 years: 64.6%  56–70 years: 59.4% | χ² (4) = 1.51 | 0.826 |
|  |  | Sex | Men: 60.0%,  Women: 61.9% | χ² (2) = 1.16 | 0.560 |
|  |  | Educational level | Low: 52.0%  Middle: 60.7%  High: 63.4% | χ² (4) = 1.62 | 0.805 |
|  |  | Professional discipline | Physicians: 45.5%  Nurses: 59.0%  Allied health professionals: 67.0%  Other: 61.2% | χ² (6) = 5.81 | 0.445 |
|  |  | Years of professional experience | 0–5 years: 66.4%  6–10 years: 55.8%  11–20 years: 70.2%  >20 years: 52.5% | χ² (6) = 10.38 | 0.110 |
|  | Students | Age group | 16–25 years: 55.3%  26–50 years: 56.0% | χ² (2) = 0.07 | 0.968 |
|  |  | Gender | Men: 54.9%  Women: 55.7% | χ² (4) = 5.03 | 0.285 |
| **Interest in education on dementia risk reduction** | Professionals | Professional discipline | Physicians: 45.5%  Nurses: 59.1%  Allied health professionals: 65.5%  Other: 50.0% | χ² (6) = 4.90 | 0.557 |
|  |  | Years of professional experience | 0–5 years: 60.3%  6–10 years: 66.7%  11–20 years: 60.3%  >20 years: 48.9% | χ² (6) = 10.45 | 0.107 |
| **Current information dissemination on dementia risk reduction** | Professionals | Years of professional experience | 0–5 years: 57.4%  6–10 years: 67.4%  11–20 years: 67.2%  >20 years: 60.2% | χ² (3) = 2.51 | 0.473 |
